# Molecular insights into kaempferol derivatives as potential inhibitors for CDK2 in colon cancer: pharmacophore modeling, docking, and dynamic analysis

**DOI:** 10.3389/fchem.2024.1440196

**Published:** 2024-08-21

**Authors:** Fei Xing, Zhicheng Wang, Noor Bahadar, Can Wang, Xu-Dong Wang

**Affiliations:** ^1^ Department of Gastrointestinal Surgery, The Second Hospital of Jilin University, Changchun, Jilin, China; ^2^ Key Laboratory of Molecular Epigenetics of the Ministry of Education (MOE), School of Life Sciences, Northeast Normal University, Changchun, Jilin, China

**Keywords:** CDK2, colorectal cancer, kaempferol derivatives, computational studies, 3D structures of protein complexes

## Abstract

Cyclin-dependent kinase 2 (CDK2) has been recognized as one of the crucial factors in cell cycle regulation and has been proposed as a potential target for cancer therapies, particularly for colorectal cancer (CRC). Due to the increased incidence rate of CRC and challenges associated with existing treatment options, there is a need for efficient and selective anti-cancer compounds. The current work aims to explore the ability of novel kaempferol derivatives as CDK2 inhibitors by performing conceptual pharmacophore modeling, molecular docking, and molecular dynamic analysis. Kaempferol and its derivatives were obtained from PubChem, and the optimized 3D structures of the compounds were generated using Maestro Ligprep. Subsequently, a pharmacophore model was developed to identify compounds with high fitness values, resulting in the selection of several kaempferol derivatives for further study. We evaluated the ADMET properties of these compounds to assess their therapeutic potential. Molecular docking was conducted using Maestro and BIOVIA Discovery Studio version 4.0 to predict the binding affinities of the compounds to CDK2. The top candidates were subjected to MM-GBSA analysis to predict their binding free energies. Molecular dynamics simulations using GROMACS were performed to assess the thermodynamic stability of the ligand-protein complexes. The results revealed several kaempferol derivatives with high predicted binding affinities to CDK2 and favorable ADMET properties. Specifically, compounds **5281642, 5318980,** and **14427423** demonstrated binding free energies of −30.26, −38.66, and −34.2 kcal/mol, respectively. Molecular dynamics simulations indicated that these ligand-protein complexes remained stable throughout the simulation period, with RMSD values remaining below 2 Å. In conclusion, the identified kaempferol derivatives show potential as CDK2 inhibitors based on computational predictions and demonstrate stability in molecular dynamics simulations, suggesting their future application in CRC treatment by targeting CDK2. These computational findings encourage further experimental validation and development of kaempferol derivatives as anti-cancer agents.

## 1 Introduction

Colorectal cancer (CRC) has the third highest prevalence rate of all cancer types worldwide, with an expected 2.4 million incidences by 2035 (Siegel et al., 2021; Sung et al., 2021). Surgery, chemotherapy or radiotherapy, immunotherapy, hormonal therapy, and pharmacotherapy are all used to treat CRC, focusing on the tumor site and stage of the disease (Hagan and Donovan, 2013). Apoptosis prevents damaged cells from developing out of balance under normal physiological conditions. However, secondary mutations in apoptosis-regulating genes may allow these cells to evade the regulatory mechanisms of apoptosis. Cancer chemoprevention is described as the use of chemical or organic compounds substances to prevent, suppress, or counteract tumorigenesis or the growth of metastatic carcinoma (Zhang et al., 2021).

Almost all modern therapeutic medicines have origins in herbal medicine, either as unaltered dietary supplements or as improved synthetic analogues (Parmar et al., 2016). Researchers are interested in screening herbal plants, isolating, identifying, and evaluating their secondary metabolites as potential drug leads for the same reason. Some flavonoids, triterpenoids, and polyphenols found in nature have been reported to trigger endoplasmic reticulum stress-induced tissue damage through apoptosis and necrosis (Sharma and Janmeda, 2017; Liu et al., 2016). Bioactive compounds have been modified to improve therapeutic effectiveness, bioavailability, and specificity, as well as a variety of features, including the implementation of some potential chemotherapeutic agents (Patridge et al., 2016; Newman and Cragg, 2012; Mishra and Tiwari, 2011).

The high cost of synthetic medicines, their adverse reactions, drug interactions, and drug resistance issues, on the other hand, prompted researchers to look for less expensive and more effective alternatives like natural bioactive compounds (Lopez and Banerji, 2017). The rational design of effective anti-cancer agents now requires the development of molecular targets associated with cancer metabolism (Collins et al., 2017; Abonia et al., 2012; Castillo et al., 2018). Protein kinases (PKs) have received much attention due to their critical role in cell survival and proliferation regulation. Oncogenic kinases have developed as possible cancer treatment targets over the last two centuries (Tsai and Nussinov, 2013). Various kinase inhibitors have made it to the commercial, with a high response rate in lung cancer treatment (Chabner, 2004). Among such kinases are Cyclin-dependent kinases (CDKs), which play a significant role in tumorigenesis and transcription control (Gridelli et al., 2011).

Cyclin-dependent kinase 2 (CDK2) is a member of the cyclin-dependent kinase family, which plays a critical role in regulating the cell cycle. It forms complexes with D-type cyclins, which subsequently phosphorylate the retinoblastoma protein (Rb). This phosphorylation event releases E2F transcription factors, thereby promoting the transcription of genes necessary for DNA synthesis and cell cycle progression. CDK2 primarily binds to cyclins A, B, and E and is essential to cell cycle regulation. It is responsible for G1 to S phase transition in the cell cycle.

CDK2 is a serine/threonine kinase composed of several critical structural domains: N-terminal Domain: This domain contains the ATP-binding site, which is crucial for the kinase activity of CDK2. The glycine-rich loop (G-loop) within this domain binds and orients ATP. C-terminal Domain: This domain includes the activation segment, which undergoes conformational changes upon binding to cyclins and subsequent phosphorylation. Catalytic Domain: The catalytic domain is located between the N-terminal and C-terminal domains. It contains the substrate-binding site where the phosphorylation of target proteins occurs. Several critical structural features characterize the binding site of CDK2. The ATP-binding pocket is located within the N-terminal domain, where this pocket binds ATP and positions it to transfer the phosphate group to the substrate. Activation Segment: This segment includes the T-loop, which must be phosphorylated for the kinase’s full activation. The positioning of this loop influences the accessibility of the substrate-binding site. Cyclin-binding Interface: This interface is essential for interacting with D-type cyclins, which is necessary for activating CDK2.

In normal healthy cells, CDK2 is dispensable as CDK1 plays mimicking roles. In cancerous cells, however, CDK2 plays a pivotal role in cell growth and progression (Shi et al., 2015; Wood et al., 2018). Overexpression of CDK2 and cyclins A and E has been observed in ovarian, colorectal, breast, prostate, and lung cancer patients (Shi et al., 2015; Tadesse et al., 2019). Therefore, drugs such as flavopiridol, roscovitine, olomoucine, adapalene, and kenpaullone, which are reported to be CDK2 inhibitors, and sorafinib, aspirin (salicylic acid), etc., which have been reported to cause downregulation of the enzyme via various mechanisms have been employed as therapies for these cancers (Oh et al., 2012; Cicenas et al., 2015; Dachineni et al., 2015).

Molecular docking is used to compute the binding affinity of ligand molecules, which is essential in understanding their pharmacological and biological activities (Lokhande et al., 2019; Rajendran and Sethumadhavan, 2013). The discovery of the protein target and its regulator is typically the first approach in searching for novel pharmacological compounds (Adewole and Ishola, 2019; Tripathi and Khan, 2018). Protein-protein interactions are significant to many biological processes, and their disruption is a leading cause of disease. The use of small molecules to modulate them is gaining popularity, but protein interfaces usually lack specific cavities for processing small molecules. In addition, since protein-ligand interactions are fundamental in drug design, the current research was investigated using molecular docking.

## 2 Materials and methods

### 2.1 Ligands

The 3-D conformers/chemical files of kaempferol and its derivative ligands were retrieved from PubChem in sdf format. Maestro Ligprep module was used to optimize the geometry and energy settings of traditional Chinese medicine compounds. The ligPrep panel is a tool used to set up preparation calculations and start the configuration preparation for that. We aimed to generate 3D structures corresponding to the input 2D framework and the low-energy isomers that Glide and other programs needed. Using Epic, we obtained possible ionized states with a pH of 7.0 ± 2.0 and formed the required tautomers. We expect to get up to 32 stereoisomers per ligand, define chirality from available 3D coordinates, and produce rings with lower energy. Finally, the compounds’ structure and energy minimization was performed with the help of the OPLS3e force field (Wahid et al., 2021).

### 2.2 Absorption, distribution, metabolism, excretion, and toxicity analysis

The selective commercial compounds were subjected to absorption, distribution, metabolism, excretion, and toxicity (ADMET) in the Qikprop module of Maestro and SWISS ADME (http://www.swissadme.ch) and ADMET Lab 2.0 (https://admetmesh.scbdd.com/) to evaluate ADMET and drug-likeness parameters (Gajjar et al., 2021). The following parameters were studied: drug physiochemical properties, octanol/water partition coefficient, aqueous solubility, GI absorption; blockage of HERG K^+^ channels; gut blood barrier permeability, brain–blood barrier; skin permeability; human serum albumin binding; the volume of distribution; CNS permeability; bioavailability score; *P* glycoprotein transportation and contraindication with other drugs, primary metabolites; CYP450 enzymes metabolism; total clearance, and toxicity studied in AMES toxicity; oral rat acute toxicity (LD_50_); hepatotoxicity and skin sensitization (Wahid et al., 2021).

### 2.3 Ligand-based pharmacophore modeling and 3D QSAR study

The Phase module of Schrodinger was used to construct the pharmacophore of a total of polyphenols having anti-cancer characteristics. Based on their pIC50 values, a total of active ligands was selected and subsequently divided into active and inactive groups. Following that, a pharmacophore model was built. The top five pharmacophore hypotheses with the highest survival scores were picked for additional study. This investigation applied a pharmacophore matching tolerance of 2, 3D quantitative structure-activity relationship (QSAR) tests, and a thorough examination of scoring functions. A gradient-convergent optimal technique was used to minimize the ligands, and they were then aligned using flexible ligand alignment.

### 2.4 Pharmacophore modeling validations

The ligands were randomly partitioned into training and test sets during the validation phase. To construct the Quantitative Structure-Activity Relationship (QSAR) model, a training set comprising 70% of the data was retained. The QSAR model was evaluated using a Partial Least Squares (PLS) factor 4. In the analysis, a grid space of size one was utilized. As a component of the validation procedure, the predicted activity of the compounds was assessed. The statistical measures used for evaluation were the squared correlation coefficient (*R* ^2^) and the variance ratio (*F*). The pharmacophore model was validated using external test chemicals. Contour plots were used to determine the specific spatial locations within the structure that had pharmacophoric requirements.

### 2.5 Protein homology modeling

The protein homology model can be employed to predict the three-dimensional topology of CDK2 by utilizing an amino acid sequence that exhibits more significant conservation than the desired structural conformation. This method enables the prediction of a more conserved amino acid sequence than the desired structural conformation. The sequence of Cyclin-dependent kinase 6 (Q00534) was obtained from UniProt.

The Expasy ProtParam tool (http://web.expasy.org/protparam/) was used to analyze various physical and chemical characteristics. Maestro v11.8 (Schrodinger suite 2018) homology modeling approach requires some discrete procedures. The template selection is conducted using the UniProt database, followed by BLAST homology searches against the NCBI PDB database, and then the sequences are aligned using ClustW. Subsequently, a model is generated based on the aligned sequences, and then the model is evaluated. If the selected protein model does not meet the requirements specified in the Protein Reliability report of Maestro 11.8, then the model was refined. The model had undergone enhancements utilizing the VSGB solvation model, OPLS3e force field in Prime Refine Loops, and Prime Minimize modules of Maestro 11.8 (Schrodinger suite 2018-4). By optimizing the loops and minimizing the energy, this modification resulted in a notable improvement in the accuracy of the protein model. The minimized model was evaluated using Ramachandran plots through PROCHECK on SAVES version 6 (http://services.mbi.ucla.edu/SAVES/), and the alignment of the template on the PDBSum server (https://www.ebi.ac.uk/thornton-srv/databases/cgi-bin/pdbsum) (Wahid et al., 2022).

### 2.6 Molecular docking

Molecular docking was performed using the various modules of Maestro and BIOVIA Discovery Studio 2021 (Schrödinger, 2021; Wahid et al., 2021; Wahid et al., 2022). The prime tool was used to fill structural gaps, and the Epik tool was employed to protonate het-groups at a pH of 7.0 ± 2.0. PROPKA optimized hydrogen bond arrangements at pH 7.0, and OPLS3e minimized constraint energy. A receptor grid box was generated using the Sitemap module, which determined the coordinates of protein binding pockets and cubic grid boxes. Molecular docking was conducted using prepared ligands, proteins, and receptor grid files. A molecular docking optimization was accomplished, extra precision docking was selected, and the Epik tool was used to apply penalties to the docking score.

### 2.7 Molecular mechanics/generalized born surface area (MM/GBSA) simulation

Molecular mechanics, general born surface area (MM/GBSA) was used to calculate the binding energies of Glide ligand-protein complexes. Prime MM-GBSA generates a lot of energy properties. These properties report energies for the ligand, receptor, and complex structures as well as energy differences relating to strain and binding, and are broken down into contributions from various terms in the energy expression. Molecular dynamics simulations: The stability of the binding complex in the docked state was assessed using GROMACS 2022.3 (http://www.gromacs.org), a widely used molecular dynamics simulation software program. Molecular dynamics simulations (MDS) were employed for this purpose. The protein’s topology was generated utilizing the GROMACS software package and the CHARMM36 force field, whereas the topology for the ligand was generated employing the Swiss-param server (https://www.swissparam.ch/). Subsequently, the protein-ligand complexes were immersed in a TIP3P water solution for solvation. We introduced sodium (Na^+^) and chloride (Cl^−^) ions to study the ligand-protein complex dissociation to achieve a physiological ionic strength environment. This setup aimed to mimic the ionic conditions present *in vivo*, which can influence the binding interactions and stability of the complex.

The ligand-protein complex dissociation was achieved by introducing sodium (Na^+^) and chloride (Cl^−^) ions at a concentration of 0.15 mM into a cubic box with dimensions of 1 nm on each side. A notable decrease in the system’s energy consumption was achieved by employing a steep-descent methodology and executing a total of 50,000 iterations. The NVT ensemble, also known as the canonical ensemble, and the NPT ensemble, also known as the isothermal-isobaric ensemble, were employed to conduct an ensemble equilibration for 200 ps. The temperature was reduced to 310 K using a modified Berendsen thermostat, while the pressure was maintained at 1 atm using the Berendsen method. The electrostatic and van der Waals interactions at a 1 nm threshold were determined using the particle-mesh-Ewald and van der Waals approaches. Following a simulation of the system for 40 ns, utilizing a time step of 2 fs, the subsequent trajectory was recorded at intervals of 10 ps. Through the examination of the simulated trajectory data, it became feasible to quantify many parameters, namely, the root mean square deviation (RMSD), root mean square fluctuation (RMSF), radius of gyration (Rg), solvent-accessible surface area (SASA), and hydrogen bond interactions (Wahid et al., 2021; Wahid et al., 2023).

### 2.8 Softwares

Molecular docking was carried out using Maestro v11.8 (Schrodinger suite 2018-4) and BIOVIA Discovery Studio 2021. MDS was performed using GROMACS 2022.

## 3 Results and discussion

### 3.1 Protein homology modelling

#### 3.1.1 Physiochemical characteristics

The software tool ProtParam was employed to assess the physiochemical properties of CDK2 sequences. Its amino acid composition consisted of 326 amino acids. The highest percentage of amino acid residues observed was 12.0% for Leu, 8.6% for Val, and 7.1% for Asp. The collective count of negatively charged residues, specifically glutamic acid (Glu) and aspartic acid (Asp) was found to be 45. In contrast, the count of positively charged residues, namely, arginine (Arg) and lysine (Lys), was determined to be 40. The protein is somewhat acidic, evidenced by its PI value of 6.02.

Furthermore, its instability index of 35.16 suggests that a lack of stability characterizes the protein. This instability was predicted due to the absence of certain dipeptides in stable proteins. The aliphatic index of the protein, which measures the relative abundance of aliphatic amino acids, is 91.08. This value indicates a moderate level of thermostability for the protein. Additionally, the protein’s GRAVY score, which quantifies the hydrophobicity of the amino acid sequence, is −0.247. This score reflects a tendency for beneficial interactions with water. At a wavelength of 280 nm, the extinction coefficient for Cysteine (Cys), Tryptophan (Trp), and Tyrosine (Tyr) concentrations is around 28,795 M^−1^cm^−1^. The coefficient above is valuable for quantitatively assessing interactions between proteins and ligands in a solution.

#### 3.1.2 Validation of homology modeling

**FIGURE 1:**
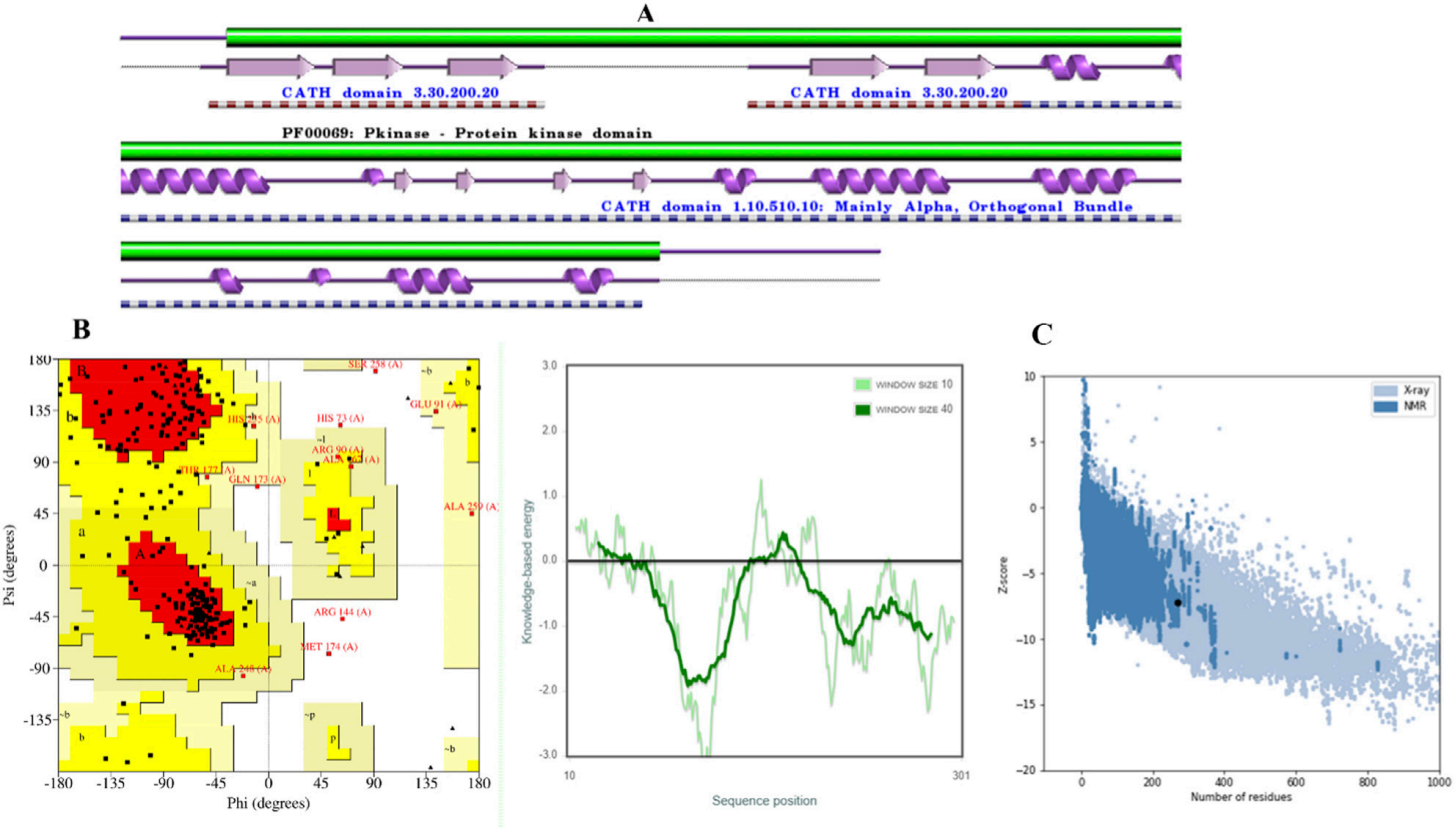
Validation of CDK2 models. **(A)** CDK2 Secondary structure. **(B)** The distribution of amino acid residue in Ramachandran plot. **(C)** Z–score and quality plots for protein models.

The homology BLAST search was employed to identify suitable templates for the homology modeling of CDK2. The results of the sequence alignment are illustrated in Figure 1A. The sequence alignment analysis of template AQ1 revealed a perfect match with 100% identity without any observable gaps.

The model was subjected to PROCHECK validation, which involved evaluating the Ramachandran plot. The plot depicted the distribution of amino acids for psi and phi angles, Figure 1B. These calculated values were found to be beneficial in determining the structure. The homology model displayed a region that was found to be favorable, with a residue count exceeding 71.4%. The investigation results indicated the absence of amino acid residues inside restricted areas. The Z-Score of the model employed for evaluating the overall quality was determined to be −7.32, Figure 1C. A suitable model can be created through molecular docking research, even in cases of limited sequence similarity, when the alignment is completed accurately.

### 3.2 Ligand-based pharmacophore model

**FIGURE 2:**
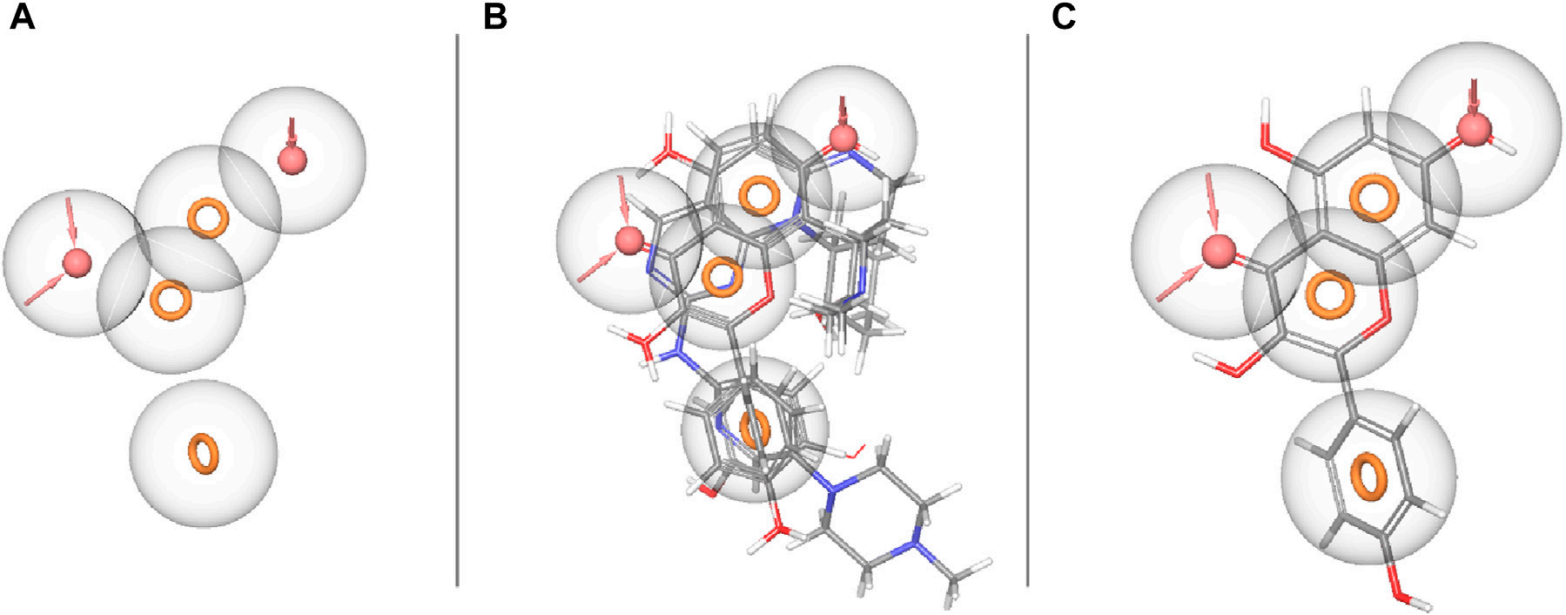
Ligand-based pharmacophore model. **(A)** Hypothesis. **(B)** Matched compounds fitted in hypothesis. **(C)** Reference ligand fitted in hypothesis.

The concept of pharmacophore encompasses the essential physical and chemical attributes, as well as the spatial organization, that are required to identify ligands by biomacromolecules. The acquisition of polyphenols that possess specific targets or exhibit similar properties can be facilitated by utilizing the screening chemical databases alongside a pharmacophore model. Pharmacophore models could be classified into two primary classifications: structure-based models and ligand-based models. The present investigation entailed developing a series of three-dimensional pharmacophore models for CDK2 by utilizing established inhibitors. The examination of the shared features of biological activity involved the alignment of these inhibitory medicines. The pharmacophore model (AARRR) that exhibits the highest optimization level consists of five unique properties: an aromatic ring, two hydrogen bond acceptors, a hydrogen bond donor, and a negatively charged ion core. This configuration is visually represented in Figure 2. The pharmacophore score of different hypothesis models is provided in Table 1. While the pharmacophore modeling of matched compounds is provided in Table 2.

**TABLE 1:** Pharmacophore score of different hypothesis models.

Sr no	Hypothesis	Phase hypo score	EF1%	BEDROC160.9	Total actives	Ranked actives	Matches
1	AARRR_1	1.02	60.6	0.8	10	7	5 of 5
2	AAAR_1	0.99	60.6	0.77	10	9	4 of 4
3	AARR_4	0.99	60.6	0.77	10	10	4 of 4
4	AARRR_2	0.98	70.7	0.79	10	7	5 of 5
5	AARR_3	0.98	60.6	0.77	10	9	4 of 4
6	AARRR_3	0.98	70.7	0.82	10	7	5 of 5
7	AARR_1	0.98	60.6	0.78	10	7	4 of 4
8	ARRR_1	0.97	60.6	0.77	10	7	4 of 4
9	ARRR_2	0.94	60.6	0.77	10	7	4 of 4
10	AARR_5	0.94	60.6	0.73	10	9	4 of 4
11	AAAR_2	0.94	60.6	0.77	10	9	4 of 4
12	AARR_2	0.94	60.6	0.77	10	7	4 of 4
13	AAARR_2	0.93	70.7	0.68	10	7	5 of 5
14	AARRR_4	0.92	60.6	0.69	10	7	5 of 5
15	AAARR_1	0.92	60.6	0.62	10	7	5 of 5
16	AARR_6	0.92	60.6	0.77	10	9	4 of 4
17	AAADR_2	0.72	20.2	0.37	10	6	5 of 5
18	AAADR_1	0.57	20.2	0.32	10	6	5 of 5

**TABLE 2:** Pharmacophore modeling of matched compounds.

Sr no	Pubchem ID	Vector score	Volume score	Matched sites matched	Matched ligand sites	Fitness	Phase screen score	Align score	Group fitness	Hypothesis
1	5281649	0.998	0.919	5	A (1) A (5) R (14) R (13) R (15)	2.892	2.892	0.03	2.892	AARRR
2	22507438	0.717	0.547	5	A (3) A (5) R (10) R (9) R (11)	1.146	1.146	1.342	1.146	AARRR
3	44258716	1	0.921	5	A (1) A (6) R (14) R (13) R (15)	2.876	2.876	0.054	2.876	AARRR
4	21596130	0.89	0.833	4	A (2) A (5) R (12) R (-) R (11)	2.273	2.273	0.06	2.273	AARRR
5	1.4E+08	0.997	0.916	5	A (1) A (6) R (14) R (12) R (13)	2.882	2.882	0.038	2.882	AARRR
6	51041990	0.995	0.747	5	A (3) A (7) R (18) R (16) R (17)	2.708	2.708	0.041	2.708	AARRR
7	5487756	0.998	0.902	5	A (1) A (5) R (12) R (11) R (13)	2.873	2.873	0.032	2.873	AARRR
8	21932272	0.992	0.892	4	A (3) A (5) R (12) R (-) R (11)	2.434	2.434	0.068	2.434	AARRR
9	21596130	0.998	0.904	4	A (2) A (5) R (12) R (-) R (11)	2.452	2.452	0.06	2.452	AARRR
10	53262726	0.998	0.878	4	A (2) A (4) R (10) R (-) R (11)	2.427	2.427	0.059	2.427	AARRR
11	5481970	0.998	0.935	5	A (1) A (5) R (12) R (11) R (13)	2.907	2.907	0.032	2.907	AARRR
12	52945930	0.746	0.509	4	A (2) A (4) R (12) R (-) R (11)	1.585	1.585	0.669	1.585	AARRR
13	176907	0.708	0.571	4	A (2) A (3) R (8) R (-) R (7)	1.572	1.572	0.735	1.572	AARRR
14	1.27E+08	0.722	0.531	5	A (4) A (5) R (11) R (9) R (10)	1.125	1.125	1.353	1.125	AARRR
15	179999	0.893	0.817	4	A (2) A (5) R (10) R (-) R (9)	2.261	2.261	0.06	2.261	AARRR
16	620596	0.931	0.821	4	A (2) A (3) R (8) R (-) R (7)	2.303	2.303	0.055	2.303	AARRR
17	1.3E+08	0.757	0.795	4	A (2) A (4) R (9) R (-) R (8)	2.103	2.103	0.067	2.103	AARRR
18	24795707	0.993	0.765	4	A (3) A (5) R (13) R (-) R (12)	2.308	2.308	0.062	2.308	AARRR
19	620596	0.998	0.875	4	A (2) A (3) R (8) R (-) R (7)	2.423	2.423	0.066	2.423	AARRR
20	12084411	0.932	0.8	4	A (2) A (5) R (10) R (-) R (9)	2.283	2.283	0.055	2.283	AARRR
21	13058505	0.972	0.861	4	A (3) A (4) R (8) R (-) R (7)	2.384	2.384	0.051	2.384	AARRR

### 3.3 Binding pocket prediction

**FIGURE 3:**
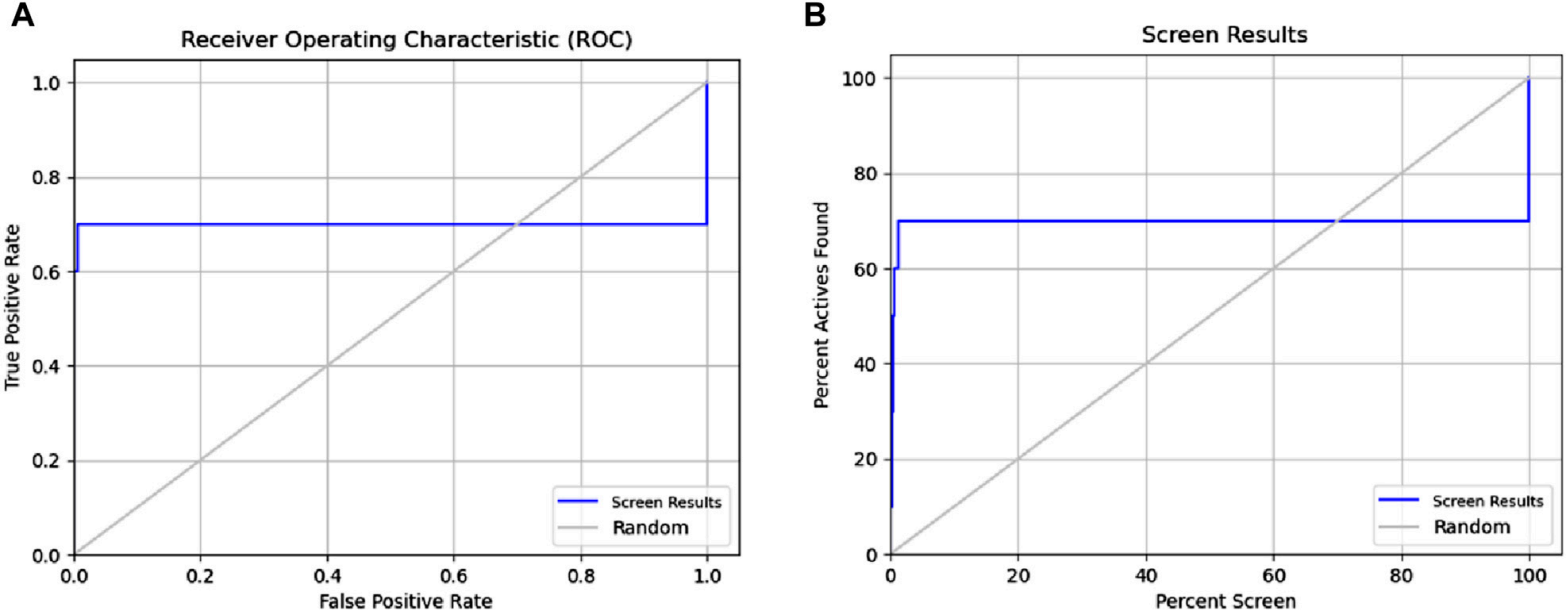
Receiver operating characteristic (ROC) and Phase Screening of Pharmacophore Model. **(A)** ROC curve analysis of the predictive model, demonstrating the true positive rate (sensitivity) against the false positive rate (1-specificity) at different threshold settings. The area under the ROC curve (AUC) provides a measure of the model’s ability to distinguish between classes. **(B)** Screening results, displaying the percentage of active compounds found against the percentage of the screened dataset.

The binding site was identified from the maestro sitemap module. Figure 3 represents the binding site and list of residues of the binding pocket. The Sitemap module provides a detailed analysis of potential binding sites based on various criteria, including size, shape, and the presence of hydrophobic and hydrophilic regions, which are crucial for ligand binding. The selection of the binding site was guided by the highest-scoring site according to the Sitemap scoring function, which evaluates the potential for favorable interactions between the ligand and the protein.

### 3.4 Molecular docking

**FIGURE 4:**
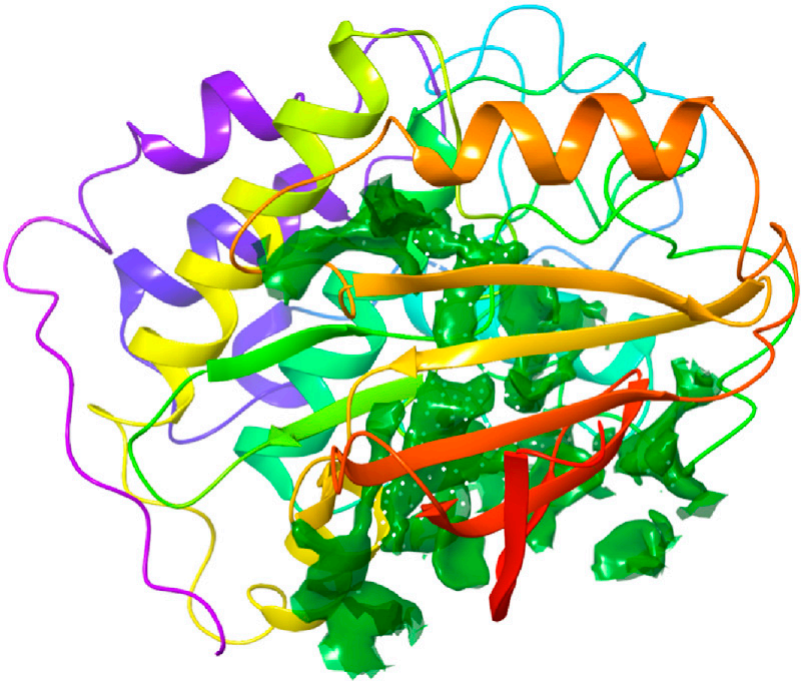
Binding site pocket with their residues of the predicted CDK2 protein retrieved from the Sitemap module.

Molecular docking was performed using the Glide module of the Schrödinger software suite (Schrödinger, 2021). The OPLS_2005 force field (Friesner et al., 2006; Halgren, 2009; Shivakumar et al., 2010) was utilized for energy optimization of the docking poses, which is well-regarded for its accuracy in predicting binding affinities and poses. The application of molecular docking plays a crucial role in the drug design process, as it enables the identification of the bioactive conformation of small and medium-sized molecules at protein binding sites and the examination of interactions between protein ligands. The Glide module utilizes an approach that systematically investigates the orientation, conformation, and spatial disposition of docking ligands to analyze molecules. The first step in the process entails minimizing the search area by use of approximate positioning and evaluation. The energy optimization of the candidate posture is subsequently performed via the OPLS-AA non-bonding potential grid. The selection and assessment of the optimal docking position is ultimately determined by applying a functional model that incorporates experiential knowledge and force field analysis. The current study focused on creating receptor grids using the Glide module, with coordinates X = −11.072, Y = 1.71, and Z = −17.085. The Maestro software was utilized to conduct molecular docking of the compounds with CDK2 to evaluate and analyze their binding affinity. Firstly, we performed the HTVS screening (Supplementary Table S1) and had a cut value of −6.5 kcal/mol; then, 122 compounds were selected for standard SP molecular docking (Supplementary Table S2). In SP docking screening, we get 21 compounds for further analysis at a −5.0 kcal/mol cut value. These 21 Compounds were screened with XP docking and then MMGBSA. The comparative study of compounds targeting CDK2 is depicted in Table 3, including their relative docking and MMGBSA scores—the binding affinities of 21 compounds mentioned in Table 3 (Figure 4; Table 3).

**TABLE 3:** Docked ligand-protein complex binding energies (kcal/mol) calculated with Prime MM–GBSA.

Sr no	Pubchem ID	Molecular weight	Docking score (kcal/mol)	Glide gscore (kcal/mol)	Glide energy (kcal/mol)	XP gscore	∆G bind (kcal/mol)	∆G bind coulomb (kcal/mol)	∆G bind covalent (kcal/mol)	∆G bind hbond (kcal/mol)	∆G bind lipo (kcal/mol)	∆G bind packing (kcal/mol)	∆G bind solv GB (kcal/mol)	∆G bind vdW
1	5281642	302.24	−11.449	−11.506	−44.683	−11.506	−46.78	−22.48	0.47	−2.93	−9.66	−0.7	24.85	−36.34
2	5318980	368.385	−10.918	−10.938	−47.878	−10.938	−52.69	−14.64	5.13	−2.01	−18.08	−1.56	23.22	−44.75
3	14427423	372.374	−10.729	−10.739	−49.353	−10.739	−54.59	−20.2	3.47	−2.69	−15.77	−1.22	26.76	−44.93
4	5281638	302.24	−10.713	−10.76	−44.708	−10.76	−49.48	−24.73	0.42	−3.51	−10.67	−0.77	25.69	−35.91
5	24857900	422.477	−10.588	−10.61	−54.886	−10.61	−62	−18.24	5.62	−2.4	−19.68	−1.42	31.11	−56.99
6	9979767	424.493	−10.568	−10.568	−51.171	−10.568	−50.08	−11.1	2.17	−1.77	−13.94	−1.44	26.41	−50.42
7	5377089	244.203	−10.541	−10.805	−35.549	−10.805	−39.42	−24.96	3.11	−2.22	−7	−0.31	19.74	−27.76
8	5281699	316.267	−10.527	−10.563	−43.808	−10.563	−40.04	−15	6.23	−2.12	−13.32	−1.52	22.08	−36.39
9	22239065	286.24	−10.518	−10.546	−42.956	−10.546	−53.83	−25.21	2.91	−1.77	−15.38	−1.18	22.09	−35.28
10	132517368	370.401	−10.321	−10.322	−46.27	−10.322	−54.64	−6.58	4.78	−1.5	−15.65	−0.96	14.54	−49.27
11	10636768	284.268	−9.97	−9.982	−41.429	−9.982	−38.93	−25.03	7.25	−2.25	−11.71	−0.76	24.77	−31.2
12	10518023	296.322	−9.922	−9.934	−38.178	−9.934	−48.13	−13.81	5.31	−1.32	−12.63	−1.08	14.9	−39.5

### 3.5 ADMET

The study utilized computational tools such as Qikprop, SWISS ADME, and ADMET Lab 2.0 to generate predictions for various physiochemical, medicinal chemistry, and ADMET parameters. These predictions were developed for polyphenols identified using an XP molecular docked compound (Table 4). Every chemical exhibited a distinct and noteworthy attribute. As a result, ADMET analysis has enabled the discovery of ligands that exhibit pharmacokinetic characteristics that meet acceptable thresholds. The pharmacokinetic characteristics of all drugs were determined to be highly favorable since no notable side effects were identified. This indicates that the compounds will likely possess good bioavailability and safety profiles based on the *in silico* models. These computational predictions suggest that the compounds have promising prospects for therapeutic applications. However, it is essential to note that these results are based on computational models and should be experimentally validated in future studies. This study analyzed twelve compounds with diverse chemical structures to evaluate their pharmacokinetic, pharmacodynamic, and toxicological properties. The molecular weights of the compounds ranged from 244.04 to 424.19, with corresponding volumes between 233.231 and 444.3. Solubility, distribution, and permeability analyses revealed significant variation among the compounds, with LogP values suggesting differences in lipophilicity and membrane permeability. The compounds exhibited high plasma protein binding, with PPB values ranging from 87.61% to 100.35%, indicating strong binding affinity. Absorption studies using Pgp inhibition and substrate values, alongside human intestinal absorption (HIA) metrics, suggested limited absorption for most compounds, corroborated by Caco-2 and MDCK permeability assays. Blood-brain barrier penetration (BBB) values highlighted some compounds’ potential for central nervous system activity. Metabolic profiling showed diverse interactions with cytochrome P450 enzymes, crucial for understanding drug-drug interactions and metabolic stability. Clearance and half-life values indicated varying elimination rates, influencing dosing regimens. Toxicological assessments, including hERG inhibition and Ames mutagenicity tests, revealed potential cardiotoxicity and mutagenicity for specific compounds. Additional evaluations for drug-induced liver injury (DILI), skin sensitization, and carcinogenicity provided insights into the safety profiles.

**TABLE 4:** ADMET analysis of XP docked 12 compounds.

Pubchem ID	5281642	5318980	14427423	5281638	24857900	9979767	5377089	5281699	22239065	132517368	10636768	10518023
LogS	−3.521	−3.315	−3.672	−3.487	−2.669	−3.137	−3.395	−3.74	−3.783	−4.058	−3.045	−3.698
LogD	0.953	2.964	2.123	1.067	3.493	3.367	2.479	2.308	1.691	2.647	2.42	2.88
LogP	2.33	4.968	3.239	2.08	6.227	6.369	2.571	2.553	2.349	4.131	2.4	4.448
Pgp-inh	0.004	0.599	0.006	0.008	0.313	0.658	0.002	0.008	0.006	0.872	0.018	0.086
Pgp-sub	0.089	0.01	0.003	0.003	0.041	0.745	0.068	0.048	0.002	0.002	0.068	0.869
HIA	0.165	0.021	0.036	0.011	0.014	0.012	0.027	0.024	0.008	0.04	0.22	0.006
F (20%)	0.996	0.008	0.012	0.743	0.951	0.966	0.579	0.032	0.051	0.004	0.999	0.261
F (30%)	1	0.315	0.417	0.993	0.993	0.837	0.997	0.977	0.927	0.011	0.998	0.988
Caco-2	−5.203	−4.883	−4.963	−5.255	−4.865	−4.907	−4.961	−5.064	−5.095	−4.759	−4.77	−4.88
MDCK	7.86 × 10^−6^	1.16 × 10^−5^	1.32 × 10^−5^	7.47 × 10^−6^	1.32 × 10^−5^	1.26 × 10^−5^	7.60 × 10^−5^	9.45 × 10^−5^	1.07 × 10^−5^	1.84 × 10^−5^	1.22 × 10^−5^	1.44 × 10^−5^
BBB	0.002	0.004	0.006	0.005	0.006	0.004	0.013	0.005	0.009	0.03	0.014	0.032
PPB	95.15%	93.59%	97.52%	97.95%	95.28%	93.02%	95.75%	96.29%	98.52%	87.61%	96.86%	100.35%
VDss	0.575	0.702	0.545	0.516	0.683	0.985	0.678	0.646	0.456	1.003	0.397	0.443
Fu	9.94%	7.93%	4.19%	8.22%	5.29%	7.37%	8.51%	8.09%	5.07%	10.57%	2.25%	1.32%
CYP1A2-inh	0.935	0.914	0.869	0.872	0.901	0.327	0.985	0.963	0.943	0.762	0.917	0.961
CYP1A2-sub	0.13	0.493	0.148	0.091	0.147	0.267	0.52	0.774	0.091	0.955	0.441	0.8
CYP2C19-inh	0.032	0.854	0.15	0.045	0.915	0.929	0.184	0.123	0.086	0.57	0.618	0.765
CYP2C19-sub	0.045	0.057	0.049	0.044	0.05	0.064	0.056	0.049	0.049	0.374	0.058	0.072
CYP2C9-inh	0.552	0.856	0.713	0.601	0.848	0.894	0.657	0.675	0.558	0.853	0.716	0.838
CYP2C9-sub	0.368	0.882	0.803	0.41	0.897	0.962	0.93	0.829	0.564	0.874	0.934	0.871
CYP2D6-inh	0.14	0.601	0.488	0.42	0.81	0.811	0.8	0.601	0.506	0.256	0.668	0.662
CYP2D6-sub	0.202	0.384	0.194	0.174	0.205	0.302	0.508	0.289	0.207	0.844	0.867	0.343
CYP3A4-inh	0.118	0.263	0.231	0.161	0.266	0.329	0.414	0.542	0.295	0.437	0.59	0.294
CYP3A4-sub	0.048	0.108	0.071	0.043	0.071	0.139	0.098	0.088	0.081	0.375	0.357	0.157
CL	7.368	6.135	6.315	6.077	7.255	16.786	4.893	7.014	7.022	3.387	10.442	1.732
T12	0.922	0.521	0.913	0.936	0.583	0.384	0.88	0.914	0.924	0.249	0.892	0.273
hERG	0.076	0.009	0.011	0.093	0.008	0.038	0.011	0.058	0.012	0.012	0.024	0.017
H-HT	0.118	0.839	0.166	0.087	0.889	0.887	0.078	0.063	0.205	0.216	0.184	0.069
DILI	0.966	0.982	0.98	0.985	0.978	0.856	0.941	0.978	0.987	0.916	0.937	0.938
Ames	0.562	0.701	0.786	0.718	0.727	0.017	0.659	0.611	0.813	0.36	0.662	0.398
ROA	0.038	0.236	0.117	0.069	0.188	0.799	0.066	0.07	0.336	0.394	0.125	0.341
FDAMDD	0.406	0.048	0.017	0.045	0.023	0.611	0.422	0.461	0.043	0.046	0.283	0.344
SkinSen	0.956	0.659	0.718	0.93	0.725	0.946	0.929	0.785	0.897	0.424	0.939	0.902
Carcinogenicity	0.103	0.09	0.059	0.097	0.267	0.145	0.118	0.036	0.304	0.137	0.549	0.16
EC	0.004	0.003	0.003	0.005	0.003	0.003	0.509	0.005	0.004	0.003	0.004	0.003
EI	0.934	0.543	0.843	0.923	0.785	0.781	0.982	0.916	0.907	0.037	0.949	0.889
Respiratory	0.129	0.26	0.054	0.058	0.135	0.915	0.226	0.136	0.072	0.097	0.075	0.63
BCF	1.054	1.253	1.014	1.021	1.237	1.278	1.039	1.061	0.993	1.794	1.123	1.57
IGC50	4.147	4.637	4.371	4.068	4.865	5.118	4.114	4.156	4.312	4.127	4.933	4.712
LC50	5.032	6.163	5.121	4.951	6.228	7.363	4.957	5.099	5.246	5.209	5.305	5.526
LC50DM	5.736	5.914	4.924	5.44	6.008	6.727	5.292	5.376	5.139	5.659	5.375	6.084
NR-AR	0.007	0.012	0.007	0.005	0.009	0.007	0.007	0.012	0.009	0.049	0.271	0.007
NR-AR-LBD	0.211	0.052	0.059	0.347	0.12	0.135	0.042	0.189	0.362	0.023	0.102	0.011
NR-AhR	0.975	0.94	0.931	0.961	0.933	0.876	0.968	0.965	0.932	0.948	0.866	0.959
NR-Aromatase	0.898	0.95	0.962	0.937	0.957	0.698	0.509	0.898	0.96	0.942	0.89	0.928
NR-ER	0.878	0.806	0.886	0.899	0.931	0.937	0.845	0.893	0.887	0.364	0.943	0.801
NR-ER-LBD	0.987	0.848	0.944	0.956	0.944	0.893	0.957	0.954	0.953	0.572	0.985	0.79
NR-PPAR-gamma	0.965	0.968	0.956	0.969	0.972	0.824	0.966	0.957	0.969	0.854	0.734	0.979
SR-ARE	0.86	0.834	0.79	0.835	0.849	0.92	0.878	0.829	0.85	0.861	0.905	0.921
SR-ATAD5	0.553	0.646	0.274	0.497	0.15	0.495	0.335	0.654	0.586	0.727	0.488	0.83
SR-HSE	0.895	0.233	0.12	0.584	0.741	0.921	0.567	0.507	0.664	0.066	0.93	0.84
SR-MMP	0.97	0.933	0.955	0.958	0.969	0.984	0.923	0.952	0.954	0.877	0.975	0.941
SR-p53	0.92	0.909	0.88	0.94	0.905	0.969	0.931	0.912	0.887	0.91	0.87	0.949
MW	302.04	368.13	372.12	302.04	422.17	424.19	244.04	316.06	286.05	370.14	284.07	296.1
Vol	282.767	375.116	369.247	282.767	441.663	444.3	233.231	300.063	273.977	377.752	282.482	308.284
Dense	1.068	0.981	1.008	1.068	0.956	0.955	1.046	1.053	1.044	0.98	1.006	0.96
nHA	7	6	7	7	6	6	5	7	6	6	5	4
nHD	5	3	5	5	4	4	3	4	4	2	3	2
TPSA	131.36	100.13	131.36	131.36	111.13	107.22	90.9	120.36	111.13	89.13	90.9	70.67
nRot	1	4	4	1	6	5	0	2	1	4	2	2
nRing	3	3	3	3	3	3	3	3	3	3	3	3
MaxRing	10	10	10	10	10	10	14	10	10	10	10	10
nHet	7	6	7	7	6	6	5	7	6	6	5	4
fChar	0	0	0	0	0	0	0	0	0	0	0	0
nRig	18	19	18	18	20	20	17	18	18	18	18	18
Flex	0.056	0.211	0.222	0.056	0.3	0.25	0	0.111	0.056	0.222	0.111	0.111
nStereo	0	0	0	0	0	1	0	0	0	0	0	0
NonGenotoxic_carcinogenicity	0	0	0	0	0	0	1	0	0	0	0	0
LD50_oral	0	0	0	0	0	0	0	0	0	0	0	0
Genotoxic_carcinogenicity_mutagenicity	0	0	0	0	0	0	3	0	0	0	0	0
SureChEMBL	1	0	0	1	0	0	0	0	0	0	0	0
Nonbiodegradable	1	1	1	1	1	1	1	1	1	0	1	1
Skin_sensitization	7	4	4	8	4	6	3	8	5	1	5	3
Acute_aquatic_toxicity	0	0	2	0	0	0	0	0	0	1	0	0
Toxicophores	2	1	1	2	1	1	2	1	2	1	2	1
QED	0.434	0.6	0.476	0.434	0.389	0.49	0.526	0.572	0.511	0.728	0.629	0.75
Synth	2.556	2.674	2.817	2.524	2.981	3.638	2.383	2.47	2.39	2.668	2.35	2.37
Fsp3	0	0.19	0.25	0	0.24	0.32	0	0.062	0	0.286	0.062	0.167
MCE-18	19	20	22	19	21	68.667	17	19	18	22	17	18
Natural product-likeness	1.522	1.775	1.793	1.559	2.154	2.13	1.441	1.545	1.281	1.162	1.14	1.069
Alarm_NMR	3	2	2	3	2	2	1	3	3	2	3	1
BMS	1	0	0	1	0	1	0	0	0	0	0	0
Chelating	1	1	1	2	1	0	0	2	2	0	1	0
PAINS	1	0	0	1	0	0	0	0	1	0	1	0
Lipinski	Accepted	Accepted	Accepted	Accepted	Accepted	Accepted	Accepted	Accepted	Accepted	Accepted	Accepted	Accepted
Pfizer	Accepted	Accepted	Accepted	Accepted	Accepted	Accepted	Accepted	Accepted	Accepted	Accepted	Accepted	Rejected
GSK	Accepted	Rejected	Accepted	Accepted	Rejected	Rejected	Accepted	Accepted	Accepted	Rejected	Accepted	Rejected
GoldenTriangle	Accepted	Accepted	Accepted	Accepted	Accepted	Accepted	Accepted	Accepted	Accepted	Accepted	Accepted	Accepted

Finally, compliance with drug-likeness rules (Lipinski, Pfizer, GSK, GoldenTriangle) affirmed the potential suitability of these compounds as drug candidates. This comprehensive analysis underscores the importance of multi-faceted evaluation in the early stages of drug development to identify promising candidates with favorable pharmacokinetic and safety profiles.

Table 4 provides a comprehensive analysis of various pharmacokinetic and pharmacodynamic properties of several drugs, including solubility (LogS), distribution (LogD, LogP), permeability (BBB, Caco-2, MDCK), enzyme inhibition and substrate status (CYP enzymes), and toxicity indicators (hERG inhibition, hepatotoxicity, carcinogenicity). These findings highlight the importance of understanding a drug’s absorption, distribution, metabolism, excretion (ADME), and potential toxicological effects to predict its efficacy and safety. Among the analyzed drugs, the top five include those with PubChem IDs 5281642, 5318980, 14427423, 5281638, and 24857900.

The top five compounds (PubChem IDs 5281642, 5318980, 14427423, 5281638, and 24857900) demonstrate varied pharmacokinetic and pharmacodynamic profiles, critical for drug development. **Compound 5281642:** This compound is moderately soluble (−3.521 LogS) and moderately lipophilic (2.33 LogP), with a high bioavailability [0.996 F (20%)] and strong plasma protein binding (95.15%). However, it has low intestinal absorption (0.165 HIA) and moderate clearance (7.368 CL), suggesting it might require enhancements in its absorption profile despite its favorable bioavailability and binding characteristics. **Compound 5318980:** Exhibiting high lipophilicity (4.968 LogP) and moderate plasma protein binding (93.59%), this compound has low bioavailability [0.008 F (20%)] and deficient intestinal absorption (0.021 HIA). Its clearance (6.135 CL) and short half-life (0.521 T12) indicate it might need structural modifications to improve absorption and bioavailability. It also has low cardiotoxicity (0.009 hERG), which benefits its safety profile. **Compound 14427423:** With poor solubility (−3.672 LogS) and moderate lipophilicity (3.239 LogP), this compound shows very high plasma protein binding (97.52%) and moderate clearance (6.315 CL). However, it suffers from deficient absorption (0.036 HIA) and high mutagenicity (0.786 Ames), posing significant challenges to its development. The low cardiotoxicity (0.011 hERG) is a positive aspect, but improvements in its mutagenicity and absorption are necessary. **Compound 5281638:** This compound has moderate solubility (−3.487 LogS) and lipophilicity (2.08 LogP), with a high bioavailability [0.743 F (20%)] and very high plasma protein binding (97.95%). Its moderate intestinal absorption (0.011 HIA) and clearance (6.077 CL) indicate potential as a drug candidate. However, the risk of drug-induced liver injury (0.985 DILI) and interactions with metabolic enzymes need careful management. **Compound 24857900:** This compound stands out with better solubility (−2.669 LogS) and higher bioavailability [0.951 F (20%)] compared to the others. It has high lipophilicity (6.227 LogP) and moderate plasma protein binding (95.28%), with significant potential for drug interactions due to its potent inhibition of cytochrome P450 enzymes. Its clearance (7.255 CL) and half-life (0.583 T12) suggest a need for optimization to balance efficacy and safety, particularly considering its moderate risk for liver injury (0.978 DILI) and low intestinal absorption (0.014 HIA).

**FIGURE 5:**
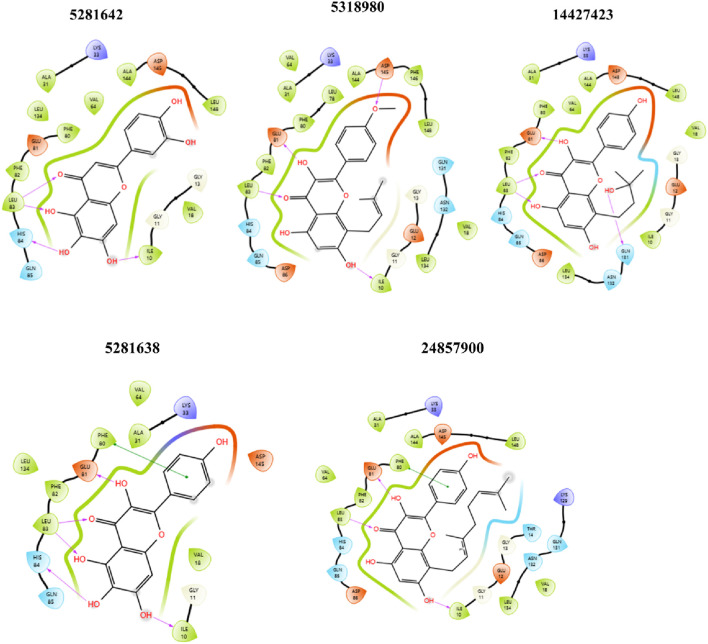
2D ligand-protein interaction between CDK2 and ligands: 5281642, 5318980, 14427423, 5281638, and 24857900. Compound 5281642: This compound forms multiple hydrogen bonds with amino acids like GLU 81, PHE 82, and ASP 145. Hydrophobic interactions are observed with LEU 83, VAL 64, and ALA 144. Compound 5318980: Similar to 5281642, this compound shows hydrogen bonds with GLU 81, PHE 80, and ASP 145. Additional interactions include those with LEU 78, ALA 144, and PHE 146. Compound 14427423: This compound forms hydrogen bonds with GLU 81, HIS 84, and ASP 145, while hydrophobic interactions are present with LEU 83, ALA 31, and VAL 64. Compound 5281638: This compound exhibits hydrogen bonding with GLU 81, PHE 82, and ASP 145, alongside hydrophobic interactions with LEU 83, VAL 64, and ALA 144. Compound 24857900: This compound forms hydrogen bonds with GLU 81, HIS 84, and ASP 86, and shows hydrophobic interactions with LEU 78, ALA 31, and VAL 64.

These compounds exhibit favorable properties such as high bioavailability and strong protein binding, alongside challenges like low absorption, potential liver toxicity, and mutagenicity. Addressing these issues through structural modifications and optimization can enhance their potential as viable drug candidates. These top five drugs, selected based on their solubility, distribution, permeability, and clearance properties, indicate their potential for further development and studyregarding efficacy and safety, as shown in Figure 5.

### 3.6 Molecular dynamic simulations

**FIGURE 6:**
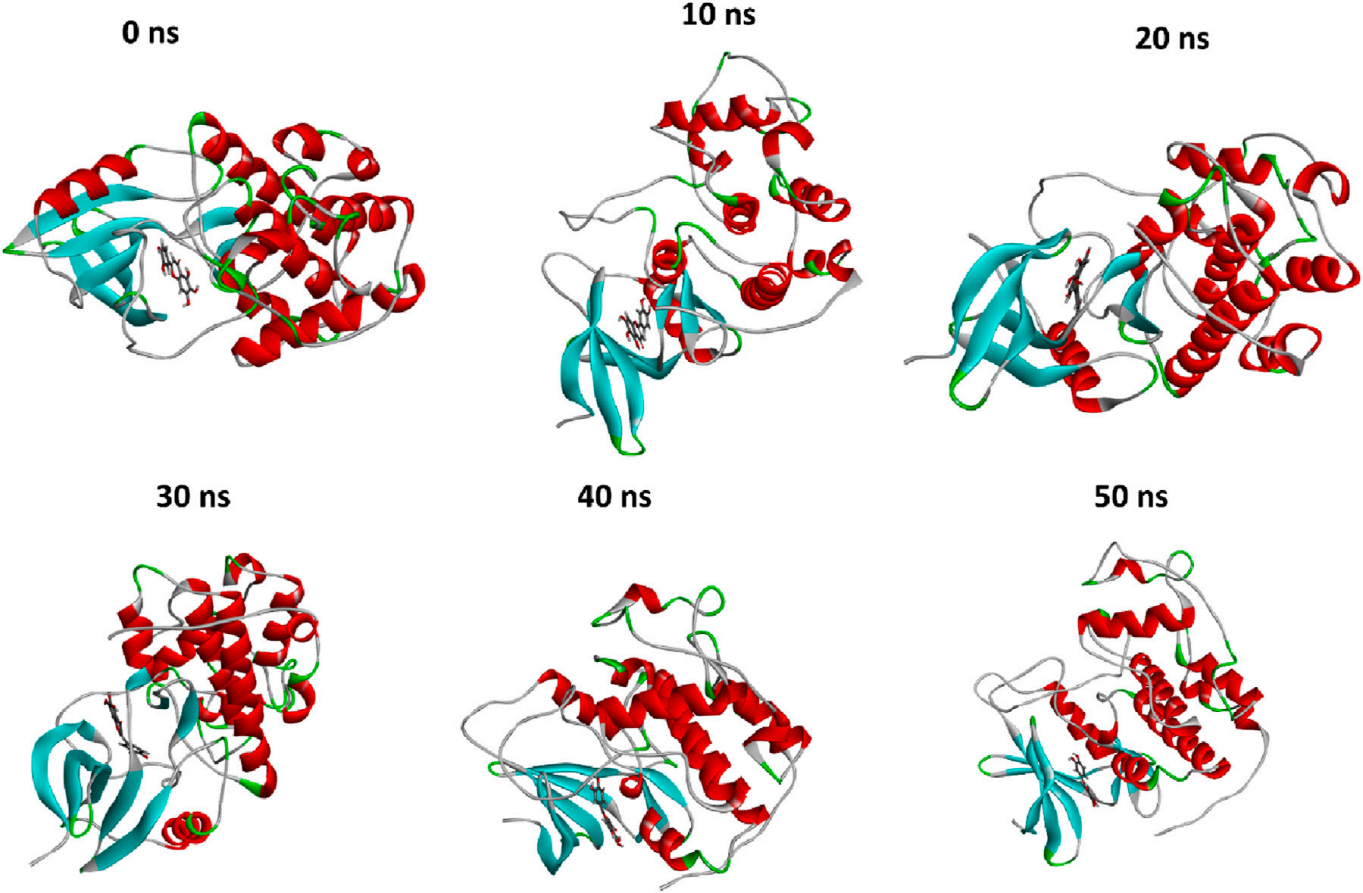
The comparison of protein conformational changes at different molecular dynamic allosteric states. At 0 ns, the initial conformation is shown, while subsequent images depict the conformational adjustments and stability of the complex over time; at 10 ns, 20 ns, 30 ns, 40 ns, and 50 ns.

MD simulation is a broadly used method in examining protein-ligand complexes, primarily focused on understanding their stability for 50 ns (Figure 6). The molecular dynamics (MD) simulation showed an observable temporal progression of binding modes within protein-ligand complexes. Within the framework of typical MD simulations, the interactions of atoms and molecules are governed by a force field. During the simulation procedure, the computation of many parameters, such as the potential energy, the total energy, the temperature, and the pressure, of the protein-ligand combination was conducted.

**FIGURE 7:**
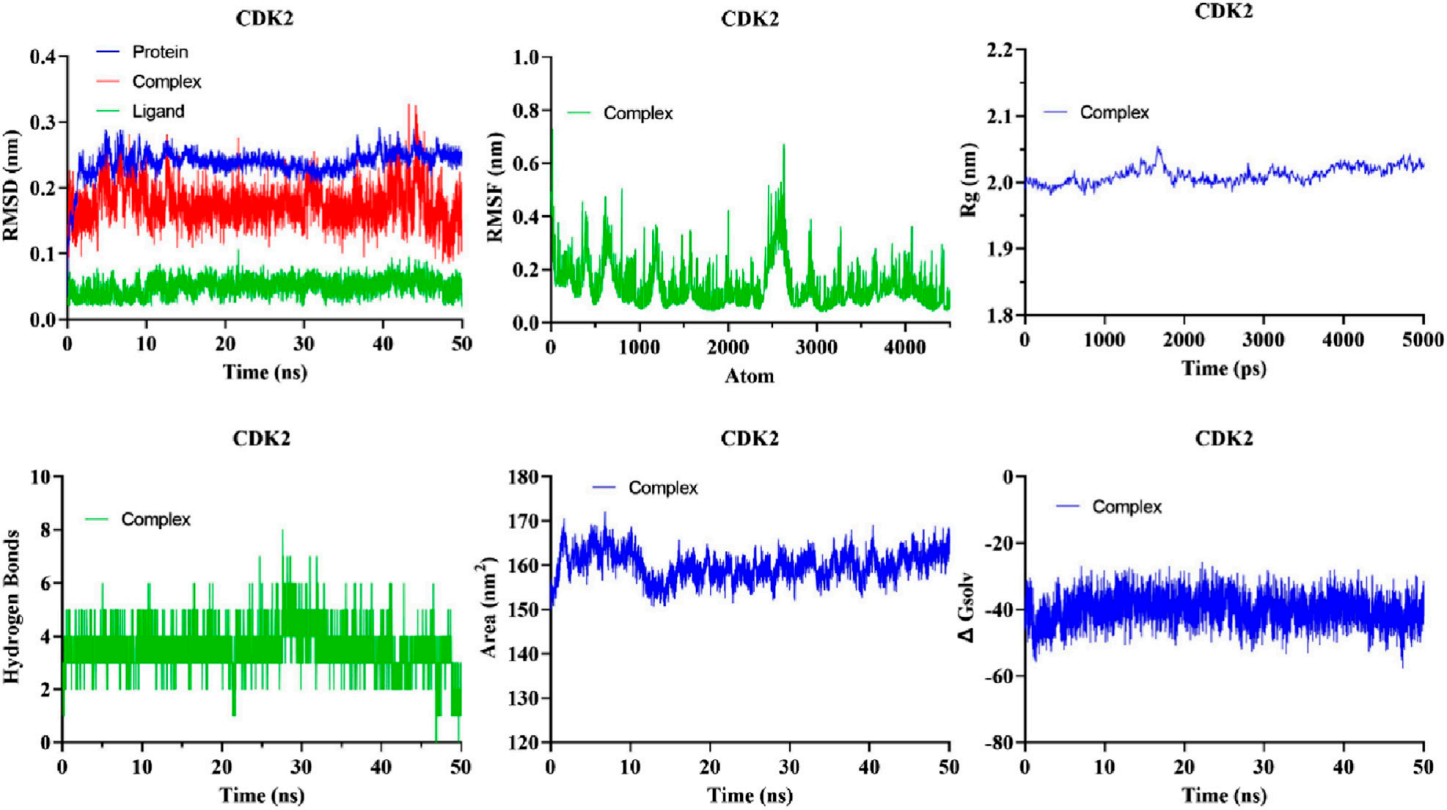
The analysis of MD simulations trajectory of docked complexes, i.e., CDK2-5281642 for 50 ns.

The evaluation of the stability of intricate chemical and protein systems, along with the stability of proteins after the introduction of small molecules and amino acid hydrophobicities, can be accomplished by quantifying molecular dynamics (MD) parameters such as RMSD, RMSF, Rg, and SASA. Based on Glide energy and ∆G binding energy we selected the 5281642 for MD simulations. The compound 5281642 showed the highest binding affinity with CDK2. Therefore, the selection of protein-ligand complexes, including 5281642, in conjunction with CDK2, was made to conduct MD simulations. The RMSD values indicated stable protein-ligand complex formation with minimal structural deviations (0.1754 ± 0.02967 nm). RMSF analysis showed low residue fluctuations (0.1512 ± 0.09623 nm), suggesting minimal conformational changes. The Rg values (2.023 ± 0.01320 nm) confirmed the compactness and structural integrity of the protein. SASA values (160.0 ± 3.225 nm^2^) and consistent hydrogen bond formation (3.403 ± 0.8496) further supported the stability of the complex. These computational findings suggest that compound 5281642 is a promising CDK2 inhibitor with potential therapeutic applications in colorectal cancer treatment, warranting further experimental validation. The results are illustrated in Figure 7. The MD simulation results demonstrate that the CDK2-5281642 complex remains stable under physiological conditions, with minimal structural deviations and maintained compactness. The low RMSF values suggest that the binding of 5281642 does not induce significant flexibility changes in the protein, and the constant Rg values indicate that the protein retains its structural integrity. Additionally, the stable SASA values imply that the ligand binding does not expose or bury significant portions of the protein’s surface area, further supporting the stability of the complex. The minimal hydrogen bond formation suggests that other non-covalent interactions play a crucial role in maintaining the strength of the ligand-protein complex.

These findings suggest that compound 5281642 is a promising CDK2 inhibitor with potential therapeutic applications in colorectal cancer treatment. The stability of the CDK2-5281642 complex in MD simulations highlights its potential for further experimental validation and drug development.

#### 3.6.1 RMSD

The quantitative assessment of the stability of the protein-ligand system is performed through the root mean square deviation (RMSD). RMSD of the protein backbone atoms in each complex exhibited a consistently low value during MD simulations (Figure 7). The mean RMSD of the docked complexes CDK2-5281642 was determined to be 0.1754 ± 0.02967 nm^2^, respectively.

#### 3.6.2 RMSF

The analytical technique called Root Mean Square Fluctuation (RMSF) enables the detection of alterations in amino acid residues within a protein during a designated temporal interval. A higher RMSF value indicates substantial fluctuations in the residues, whereas a lesser RMSF value indicates reduced levels of volatility in the residues. The impact of curcumin and EGCG on the variability of individual protein residues was evaluated by determining the RMSF of backbone atoms for each residue within the protein complex. The mean RMSF values of the CDK2-5281642 docked complexes were 0.1512 ± 0.09623 nm, respectively (Figure 7).

#### 3.6.3 Radius of gyration (Rg)

The gyratory radius is a critical measure used to assess the level of compactness shown by protein structures. Proteins with a reduced gyratory radius exhibit a more compact conformation, indicating a higher degree of stability in their structural arrangement. The average Rg values for the docked complexes of CDK2-5281642 were determined to be 2.023 ± 0.01320 nm, respectively. The lack of substantial deviations in the trajectory of the radius of gyration indicates the absence of abnormal behavior (Figure 7).

#### 3.6.4 Solvent-accessible surface area (SASA)

The SASA metric quantifies the degree of hydrophobicity a protein shows, whereby larger SASA values correspond to increased protein content and less temporal variability during simulation. Proteins can undergo structural modifications by adding small chemicals, which can induce substantial alterations in the solvent-accessible surface area (SASA). The complexes formed by the compound-protein interaction exhibited a predominantly stable behavior throughout the 40-ns simulation. The average solvent-accessible surface areas (SASA) of CDK2-5281642 complexes were determined to be 160.0 ± 3.225 nm^2^, respectively, (Figure 7).

#### 3.6.5 Hydrogen bond

The formation of hydrogen bonds considerably enhances a ligand’s capacity to interact with a protein and be recognized by the active site. During a 40-ns MD simulation, the gmx_hbond module in GROMACS was employed to analyze the number, length, and angle of hydrogen bonds within the protein-ligand complexes. The mean quantity of hydrogen bonds formed throughout the simulation was determined to be 3.403 ± 0.8496 for the CDK2-5281642 complex (Figure 7). This indicates that while the ligand forms prominent hydrogen bonds with the protein, the interaction is predominantly stabilized by other non-covalent interactions, such as hydrophobic and van der Waals forces. CDK25281642.

#### 3.6.6 Principal component analysis and free energy landscape (FEL)

**FIGURE 8:**
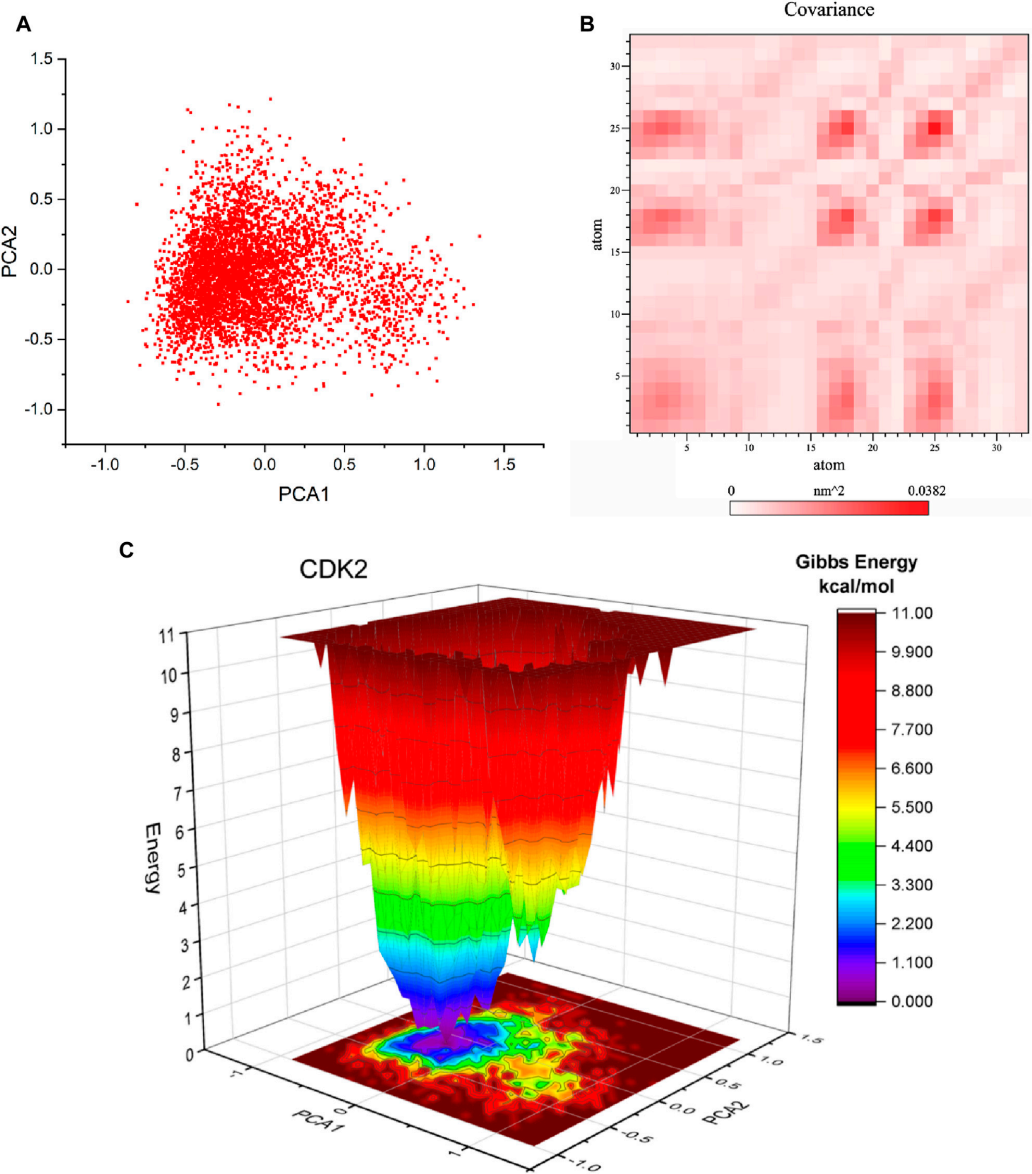
Principal component analysis (PCA), Covariance, and free energy landscape (FEL) of CDK2-5281642 docked complexes for 50 ns. **(A)** 2D PCA plots. **(B)** Covariance plots for residues of proteins. **(C)** 3D FEL of docked complexes.

Principal component analysis (PCA) is a commonly employed methodology for reducing the dimensionality of extensive datasets to extract pertinent information. Principal component analysis (PCA) was used to investigate the mobility modes of docked complexes consisting of CDK2 and its ligands 5281642. The docked complexes of CDK2-5281642 are depicted in Figure 8A using principal component analysis (PCA) plots. The implementation of routine binding procedures resulted in the formation of robust complexes. A research investigation was conducted to examine the dynamic covariance matrix of docked protein complexes to identify residues associated with demonstrating anti-correlated motion. Figure 8B displays the covariance matrix of the complexes formed by CDK2-5281642. The utilization of variations in color intensity serves as a visual representation of the correlation coefficient, a statistical measure that ranges from −1 to 1.

Free energy landscape analysis investigated the low energy conformations in the test systems involving CDK2-5281642. The generation of free energy graphs in three-dimensional (3D) and two-dimensional (2D) formats was accomplished by utilizing the first and second fundamental components. The free energy landscapes of CDK2-5281642 are depicted in Figure 8C. The free energy landscape shows energetically favorable conformations as blue dots, whereas energetically unfavorable conformations are represented by red dots. The free energy landscape of the CDK2-5281642 complex exhibits a higher concentration of blue dots, indicating that the interaction between rutin and the active site of CDK2-5281642 resulted in an energy reduction.

## 4 Key findings and conclusion

In this study, we explored the potential of novel kaempferol derivatives as inhibitors of Cyclin-dependent kinase 6 (CDK2), a crucial factor in cell cycle regulation and a promising target for colorectal cancer (CRC) therapy. Through comprehensive computational analyses, including pharmacophore modeling, molecular docking, and molecular dynamics (MD) simulations, we identified several kaempferolderivatives that exhibit high binding affinity and stability with CDK2. Our findings indicate that compound 5281642, notably, demonstrated the highest Glide energy and ΔG binding energy, making it a prime candidate for further investigation. The MD simulations revealed that the CDK2-5281642 complex remained stable under physiological conditions, with minimal structural deviations, low residue fluctuations, maintained compactness, stable solvent-accessible surface area, and consistent hydrogen bond formation. The *in silico* studies identified several kaempferol derivatives as potential CDK2 inhibitors, with compound 5281642 showing the highest binding affinity. Molecular dynamics (MD) simulations confirmed the stability of the CDK2-5281642 complex, exhibiting minimal structural deviations. MM-PBSA calculations detailedly assessed binding affinities, highlighting dominant van der Waals and electrostatic interactions. Key residues involved in stabilizing the complex included Lys33, Asp145, Phe80, and Val64. The *in silico* studies enabled the efficient identification of promising CDK2 inhibitors and provided insights into molecular interactions and structural optimization, guiding the rational design of more potent inhibitors. These findings offer a robust foundation for experimental validation, suggesting that compound 5281642 and its optimized derivatives could be effective in colorectal cancer therapy, thus streamlining the drug development process by reducing extensive trial-and-error experimentation.

The significance of this work lies in its contribution to identifying potential CDK2 inhibitors with favorable pharmacokinetic properties, which could serve as promising therapeutic agents for CRC. The computational predictions provide a robust foundation for future experimental validation, which is crucial for developing effective anti-cancer drugs. This study advances our understanding of CDK2 inhibition and its application in cancer therapy by identifying and validating novel kaempferol derivatives. These findings encourage further experimental studies to confirm the therapeutic potential of these compounds, ultimately contributing to improved treatment options for CRC patients.

## Data Availability

The original contributions presented in the study are included in the article/Supplementary Material, further inquiries can be directed to the corresponding author.
