# Supplementary material for: Molecular insights into kaempferol derivatives as potential inhibitors for CDK2 in colon cancer: pharmacophore modeling, docking, and dynamic analysis
Source: Front Chem. 2024 Aug 21;12:1440196. doi: 10.3389/fchem.2024.1440196 (PMC11371583; doi:10.3389/fchem.2024.1440196)
Supplement: Supplementary file 1 [file Image1.pdf]

Molecular Insights into Kaempferol Derivatives as Potential Inhibitors for CDK2 in Colon Cancer: Pharmacophore Modeling, Docking, and Dynamic Analysis

Fei Xing[1] xingfei21@mails.jlu.edu.cn, Zhicheng Wang[1] wangzc2024@126.com, Noor Bahadar[2] noor100@nenu.edu.cn, Can Wang[1] canwang22@mails.jlu.edu.cn, Xu-Dong Wang[1*] wangxud@jlu.edu.cn

Supplementary Data

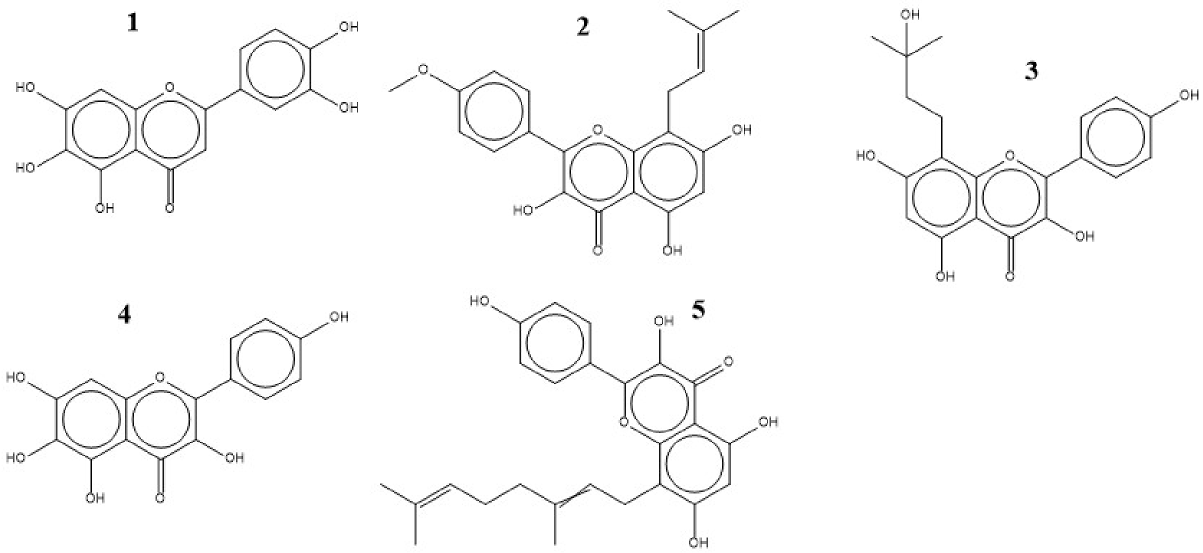

Figure S1. (1)5281642, **(2)** 5318980, **(3)**14427423, **(4)**5281638, and **(5)**24857900
